# Factors influencing behavioural intention to use a smart shoe insole in regionally based adults with diabetes: a mixed methods study

**DOI:** 10.1186/s13047-019-0340-3

**Published:** 2019-05-20

**Authors:** Emma M. Macdonald, Byron M. Perrin, Nerida Hyett, Michael I. C. Kingsley

**Affiliations:** 1 La Trobe Rural Health School, College of Science, Health and Engineering, La Trobe University, Bendigo, Australia; 2 Diabetes Centre, Goulburn Valley Health, Shepparton, Australia

**Keywords:** Diabetes mellitus, Foot ulceration, Peripheral neuropathy, Smart insole, Wearable technology

## Abstract

**Background:**

Smart insole technologies that provide biofeedback on foot health can support foot-care in adults with diabetes. However, the factors that influence patient uptake and acceptance of this technology are unclear. Therefore, the aim of this mixed-methods study was to use an established theoretical framework to determine a model of psychosocial factors that best predicts participant intention to use smart insoles.

**Methods:**

Fifty-three adults with diabetes from regional Australia completed the validated Unified Theory of Acceptance and Use of Technology (UTAUT) questionnaire. Multiple regression analysis was used to determine the psychosocial factors that best predict behavioural intention to adopt a smart insole. Additionally, a focus group was conducted and thematic analysis was performed to explore barriers and enablers to adopting this technology.

**Results:**

The multiple regression model that best predicted intention to adopt the smart insole (adjusted R^2^ = 0.51, *p* < 0.001) identified that self-efficacy (β = 0.67, *p* = 0.001) and attitude (β = 0.72, p < 0.001) were significant predictors of behavioural intention, while effort expectancy (β = − 0.52, *p* = 0.003) and performance expectancy (β = − 0.40, *p* = 0.040) were moderating factors. Thematic analysis illustrates the importance of attitude and self-efficacy on participants’ behavioural intentions, influenced by participant’s belief in the device’s clinical efficacy and anticipated effort expectancy.

**Conclusions:**

This mixed-methods study demonstrates that attitude, self-efficacy, performance expectancy and effort expectancy combine to predict intention to adopt smart insole technology. Clinicians should consider these psychosocial factors when they prescribe and implement smart soles with patients at high risk of foot ulceration.

**Electronic supplementary material:**

The online version of this article (10.1186/s13047-019-0340-3) contains supplementary material, which is available to authorized users.

## Background

Diabetes-related foot disease (DFD) is defined as ulceration, infection, ischaemia or neuro-arthropathy of the foot in people with diabetes [1, 2]. DFD generally develops from trauma in the presence of peripheral neuropathy or peripheral arterial disease and can be complicated by infection [1–4]. The frequency and severity of DFD can vary from region to region, and can be related to differences in socioeconomic conditions, type of footwear, and standards of foot care [1, 2].

Geographical rurality and remoteness are associated with increased risk of diabetes-related foot disease due to a higher disease burden and reduced access to traditional preventative services [5]. Lack of access to traditional standard preventative services is particularly important as diabetes foot disease (DFD) is often characterised by poor identification of foot-health problems, reduced self-management skills and delayed access to care [5–7]. Potential exists for information communication technologies (ICT) to overcome these geographical barriers to care.

Information communication technology has been increasingly implemented in the care of people with diabetes to improve patient outcomes in areas such as blood glucose management, diet, medication, and exercise monitoring [8–11]. The majority of trials investigating the utility of ICT for diabetes self-management have primarily focused on demonstrating the clinical efficacy of systems to reduce blood glucose levels, which has been confirmed in systematic reviews [9, 10]. However, many of these trials have been of relatively short duration and the long-term effect of ICT on health outcomes is less certain [8]. The use of ICT in the prevention and management of DFD has only recently been investigated, with studies using wearable devices, such as smart insoles, and non-wearable devices, such as a smart mat, for self-monitoring to prevent neuropathic foot ulceration being reported [12–14]. While demonstrating promise, this research is in its infancy. Mobile health technologies include portable products that gather input from separate medical devices and connect to a mobile interface [15]. In the case of smart insoles, the user receives feedback about foot-specific parameters (e.g., plantar pressures) transmitted from an instrumented insole to a mobile interface, such as a smart phone or a smart watch [13].

Little serious attention has been given to the general or specific enablers and barriers that explain the adoption and utilisation of ICT for the management of diabetes [8–11]. Consequently, a significant knowledge gap exists to explain the factors that influence adoption and usability of ICT in diabetes self-management, and for management of foot monitoring in particular. Nevertheless, extensive research regarding technology adoption has been conducted in commercial and educational settings, where there has been significant theoretical research interest in understanding issues of user acceptance [16–19]. For example, Venkatesh et al. [16] completed a systematic analysis and synthesis of eight individual technology adoption theories to develop the Unified Theory of Acceptance and Use of Technology (UTAUT). The UTAUT model articulates the way in which key psychosocial domains interact to influence behavioural intention to adopt a technology [16]. More recently, this model has been adapted for use with providers and consumers of healthcare, [17, 18] and shows potential as a theoretical underpinning in the area of adoption of ICT for diabetes self-management.

In order to determine if people with diabetes are likely to adopt ICT in the form of smart insoles to help prevent DFD, it is important to understand the psychosocial factors that might impact their intentions to use the technology.

## Methods

### Study design

The aim of this study was to determine the psychosocial factors that influence behavioural intention to adopt smart insole technology to prevent DFD for people in regional areas.

A mixed methods explanatory sequential design was undertaken [20]. The current study design is informed by Morse’s [21] definition, where mixed methods research requires the use of more than one methodological approach for the same inquiry with integration of the analysis in order to answer the one research inquiry. Ethics approval was obtained from XXXXXXXXXX and XXXXXXX University Human Research Ethics Committees (XXXX 13/16). All adults with diabetes who were enrolled in one of three outpatient podiatry programs at XXXXXX Health were identified using an administrative database and invited via letter to attend XXXX to complete the UTAUT questionnaire. Participants were invited to complete the questionnaire twice, one week apart, to determine test-retest reliability.

Participants were required to have diabetes, be English language speakers, be ambulant and able to attend the main campus in a regional city. Participants were not required to be currently engaged with any particular type of technology. Demographic data were collected for all participants, as was key information about disease status (diabetes type, duration, and history of foot ulceration).

Quantitative results derived from the UTAUT questionnaire were further investigated in a focus group, to explore meaning and to contextualise the findings [20, 21].

### Quantitative phase

The validated UTAUT questionnaire [17] was used to identify the key psychosocial factors that influence behavioural intention to adopt smart insole technology to prevent DFD in people in regional areas. For the purposes of this study, the terminology of the UTAUT questions were modified to reflect the technology type of smart insole (Table 1; Additional file 1). The questionnaire included a written description of the characteristics of a smart insole. Additionally, a verbal explanation of a smart insole was provided by the researcher administering the informed written consent process.

**Table 1:** Example of UTAUT questionnaire

Psychosocial factor	Items	Example item
Performance Expectancy	4	I would find smart insole equipment useful in managing my health.
Effort Expectancy	4	I expect to find the smart insole equipment easy to use.
Attitude	3	Using a smart insole is a good idea.
Social Influence	4	People who are important to me think that I should use smart insole equipment.
Self Efficacy	3	I could complete most tasks using smart insole equipment if there was no one around to assist me.
Anxiety	4	I would hesitate to use smart insole equipment for fear of making mistakes that I cannot correct.
Facilitating Conditions	4	I have the knowledge necessary to use smart insole equipment.
Behavioural Intentions	3	I intend to use smart insole equipment in the next 365 days.

The UTAUT questionnaire contains seven psychosocial domains and one behavioural domain incorporating a total of 29 questions measured on a 5-point Likert Scale ranging from 0 as strongly disagree to 5 as strongly agree. There are also four semi open-ended questions included. The seven identified psychosocial domains were performance expectancy, effort expectancy, social influence, facilitating conditions, self-efficacy, attitude and anxiety. The eighth domain, behavioural intention, was the outcome measure. A total score for each domain was calculated using the Likert scale.

A detailed description of the psychosocial domains has been previously provided [17]. In the context of this study, performance expectancy is the degree to which the individual believes that the smart insole will help them to prevent foot ulceration. Effort expectancy is how easy the individual believes that a smart insole will be to use in order to monitor their feet. Social influence is the degree to which significant others (e.g., family members, allied health, nurses, general practitioners) will support the adoption of a smart insole. Self-efficacy is the degree to which the individual believes that they have the skills to adopt a smart insole. Facilitating conditions refer to the degree to which the individual believes that they have the physical and cognitive capacity and infrastructure required to use a smart insole. Attitude is the individual’s positive or negative feelings towards using the smart insole. Anxiety is the self-reported degree of anxiety or hesitation a person experiences in relation to using a smart insole. Behavioural intention is the individual’s intention to use a smart insole within a twelve month period.

### Statistical methods

Quantitative statistical analyses were performed using IBM SPSS Statistics for Windows (Version 24.0; IBM Corporation, NY) with significance set at *p* < 0.05. Ratio data were presented as mean and standard deviation, and nominal data as proportions. Chi square and independent samples t-tests were used to assess differences in demographic characteristics between participants completing the questionnaire once versus twice. Cronbach’s alpha was used to assess the internal consistency of the UTAUT at baseline. Two-way mixed effects intraclass correlation co-efficient (ICC) analysis was used to evaluate test-retest reliability. For internal consistency, Cronbach’s alpha values were interpreted as follows: α < 0.70 (weak), α = 0.70–0.80 (acceptable), α > 0.80 (strong). For test-retest reliability ICC values were interpreted as follows: r < 0.50 (poor), r = 0.50–0.75 (moderate), r = 0.75–0.90 (good) and r > 0.90 (excellent) [22]. Using baseline mean UTAUT domain scores, multivariate regression analysis was performed to determine the key psychosocial domains that influence behavioural intention. Using the Enter method excluding cases pairwise, the seven psychosocial domains of the UTAUT were entered into the analysis to determine the strongest model to explain behavioural intention to use smart insoles. The required sample size was calculated in G*Power v3.1.6 using a multiple regression model with 7 predictors [23]; a minimum sample size of 49 was required to determine a true f^2^ of 0.35 (large effect) with *p* ≤ 0.05 and power of 80%.

### Qualitative phase

#### Focus group

Survey respondents were asked to indicate if they were interested in participating in a focus group. Of the survey participants who expressed their interest, ten people who represented a mix of genders, age, diabetes type and technological experience were invited to participate via letter. Six accepted the invitation, with one person cancelling due to illness. The focus group had five participants (*n* = 4 men) aged 32 to 70 years, three with a history of previous foot ulceration, four with type 2 diabetes and four with current access to the internet.

The focus group was conducted at XXXX by the first author. A semi-structured question schedule was used to explore the quantitative results and to allow the opportunity for new information to emerge. Focus group questions included “How do you feel about using an insole that can measure things, such as foot pressure and steps taken, to help you care for your feet?” The focus group was audio-recorded and transcribed verbatim using ExpressScribe (5.88; NCH Software, USA). Written field notes of reflections and observations were recorded during and immediately after the focus group for data triangulation [24].

The transcript and researcher field notes were imported into NVivo for Mac (version 11.4.1(2079) 1999–2016; QRS International Pty Ltd., Australia) for qualitative thematic analysis. Line by line coding was completed independently by two authors through initial preliminary broad-brush coding and followed by coding using a-priori thematic categories from the UTAUT domains. Codes were compared and discussed to develop themes. Disagreements were resolved by discussion to achieve consensus on the final key themes. This coding process was informed by Saldana’s descriptive coding method [25], which aims to code data to identify and describe the meaning of the text. Codes were grouped into categories, which were further analysed to develop themes that could be used to explain and understand the statistical findings. Any codes that did not align with UTAUT domains were included as additional themes.

The objective of this quantitative dominant, mixed method study (i.e., QUANT-Qual) was to obtain qualitative data that could be used to further understand the quantitative data [21]. Participants were recruited to the focus group based on key socio-demographics. Data collected during the focus group were sufficient to understand the quantitative findings indicating a-priori thematic saturation [26] and the researchers agreed that no further focus groups were required to address the aims of the study.

## Results

### Quantitative phase

**Fig. 1:**
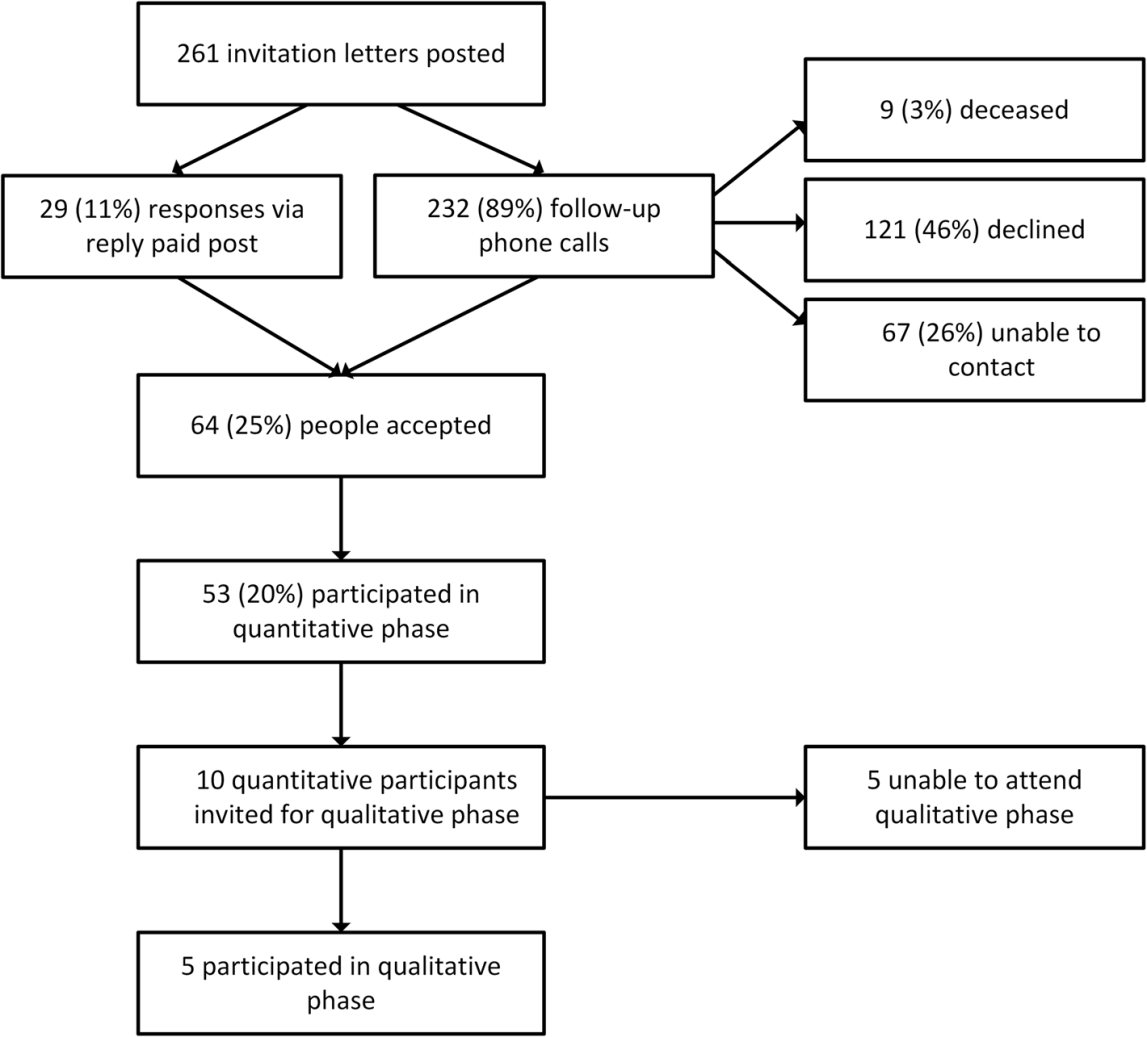
Flow diagram of the quantitative and qualitative phases of the study

Two hundred and sixty-one people were invited to participate in the study of which 53 enrolled and completed the UTAUT questionnaire at baseline (Fig. 1). The 53 participants had a mean age of 61(12.6) years of age, with the majority of participants Australian born men and with type 2 diabetes (Table 2).

**Table 2:** Participant characteristics (baseline completion of UTAUT)

Participant characteristics	Baseline measurements
Age, years	62.5 ± 10.5
Sex, men	36 (68)
Type 1 diabetes	12 (23)
Duration of diabetes, years	18.0 ± 10.4
Internet access	39 (74)
History of foot ulcers	27 (51)
Australian born	45 (85)
Completed High School	33 (62)
UTAUT domain, scores	
Performance Expectancy	3.27 ± 0.68
Effort Expectancy	3.16 ± 0.74
Attitude	3.28 ± 0.73
Social Influence	2.87 ± 0.93
Self Efficacy	3.11 ± 0.89
Anxiety	2.80 ± 1.19
Facilitating Conditions	3.22 ± 0.76
Behavioural Intentions	2.59 ± 1.17

Data are presented as mean ± SD or number (%). No significant statistical difference was found between participants who completed the questionnaire twice to those who completed the questionnaire at baseline

Thirty-two of the 53 completed the UTAUT questionnaire after one week to determine reliability. At baseline the internal consistency analysis of the domains of the UTAUT was excellent for behavioural intention (α = 0.95) and anxiety (α = 0.91), good for self-efficacy (α = 0.89), effort expectancy (α = 0.88), facilitating conditions (α = 0.88), performance expectancy (α = 0.85) and social influence (α = 0.85), and acceptable for attitude (α = 0.77). Behavioural intention displayed good test-retest reliability (r = 0.78, *p* < 0.001), while test-retest reliability of the psychosocial domains was strongest for effort expectancy (r = 0.82, *p* < 0.001) and weakest for social influence (r = 0.55, *p* = 0.020).

Significant positive correlations existed between behavioural intention and self-efficacy (r = 0.49, *p* < 0.01), attitude (r = 0.49, p < 0.01), and social influence (r = 0.37, *p* < 0.05), while anxiety (r = − 0.44, p = 0.020) was negatively correlated with behavioural intention. Effort expectancy was positively correlated with self-efficacy (r = 0.67, p < 0.001) and attitude (r = 0.58, p < 0.001). Performance expectancy was also positively correlated with self-efficacy (r = 0.50, p < 0.001) and attitude (r = 0.75, p < 0.001) (Table 3).

**Table 3:** Results from bivariate and multiple regression analyses with Behavioural Intention

	Bivariate correlation with Behavioural Intention	Multiple regression to predict Behavioural Intention			
Psychosocial domains	(r)	Standardised regression coefficient (β)	Strongest model (β)	Model adjusted R^2^	SEE
Performance Expectancy	0.27	− 0.40^*^	−0.40	0.51^**^	0.82
Effort Expectancy	0.13	−0.52^*^	−0.52		
Attitude	0.49^**^	0.72^**^	0.72		
Social Influence	0.37	0.10			
Self Efficacy	0.49^**^	0.67^**^	0.67		
Anxiety	−0.44	−0.25			
Facilitating Conditions	0.25	−0.24			

*SEE* standard error of estimate; ^*^p < 0.05; ^**^p < 0.01

**Fig. 2:**
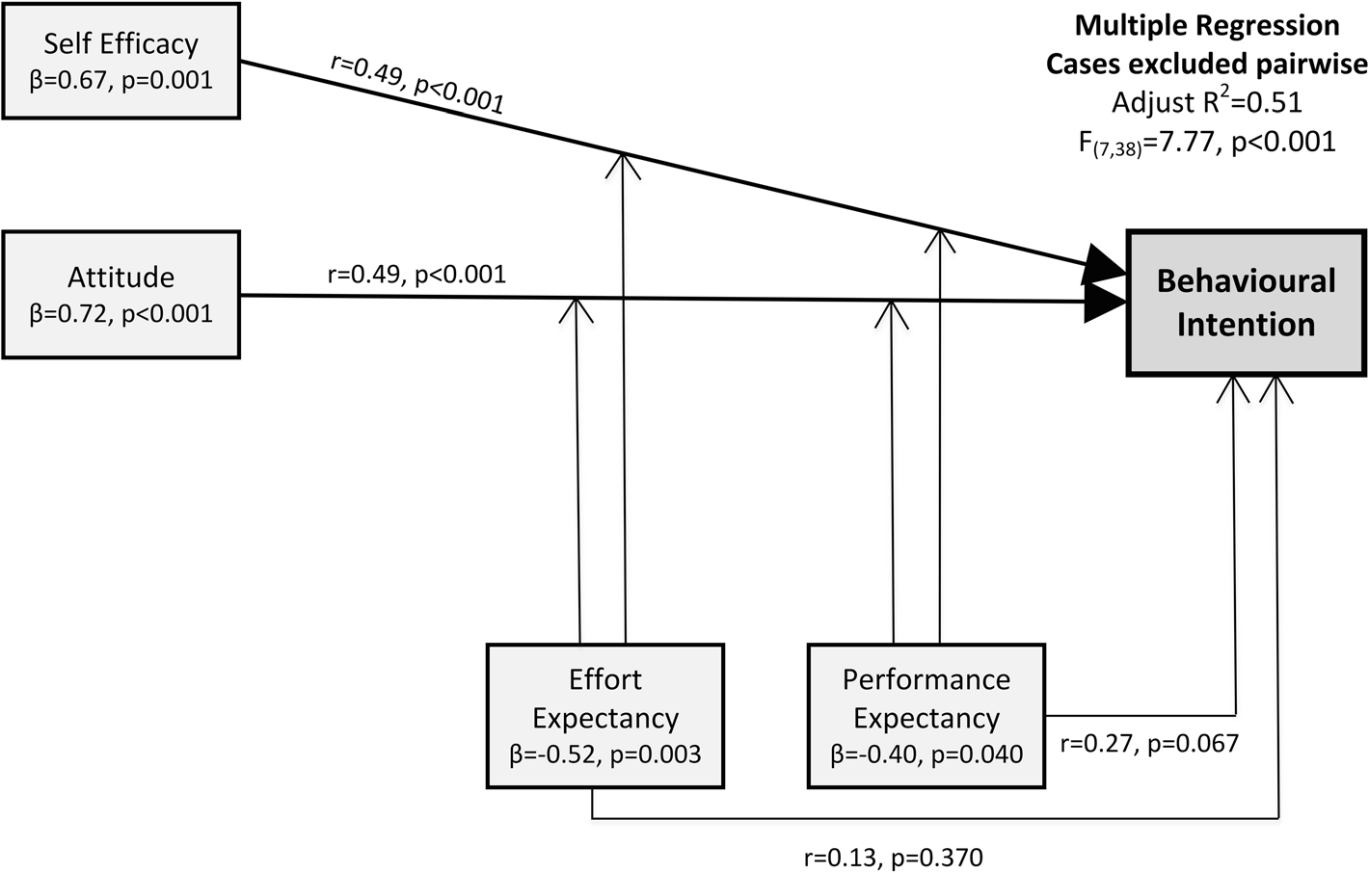
Multiple regression model

The interaction of attitude, self-efficacy, performance expectancy and effort expectancy produced the strongest regression model, explaining the majority of the variance in participants’ behavioural intention (adjusted R^2^ = 51%, p < 0.001). In this model, self-efficacy and attitude were both predictors of behavioural intention, while effort expectancy and performance expectancy acted as moderators of participants’ intention to adopt a smart insole via their impact on self-efficacy and attitude (Table 3; Fig. 2).

Quantitative content analysis of the open-ended question responses of the UTAUT revealed that 81% of participants wanted to use the insole. The most commonly cited reasons related to areas of performance expectancy such as the desire to prevent foot ulcerations, to assist with daily foot checks and an expectation that use of the insole would improve quality of life. Of the participants who did not want to use the insole, reasons included the belief that it would not help them, not having previously used the technology, and concerns about its cost. Only 15% of respondents reported anxiety about using the system, however they felt that with training they would be able to use it.

### Qualitative phase

Themes developed from thematic analysis of the focus group transcript support and further explain the model presented in Fig. 2 and the semi open-ended questionnaire responses. Focus group findings illustrate how attitude and self-efficacy interact with performance expectancy and effort expectancy to influence participants’ intention to adopt a smart insole (Table 4).

**Table 4:** Focus group themes

Focus Group Theme	Participant Quote
Attitude towards technology “Bring it on!”	*“Well as far as I am concerned it’s actually probably three years too late.… within eight weeks of that diagnosis I’d lost my left leg. Now theoretically if that information had been available 6 months earlier, downloaded and gone to my GP, there would have been red alerts everywhere.” (P1)* *“…if it was available, I’d use it to prevent, because we know an ounce of prevention is better than a pound of cure.” (P2)* *“..I think it’s very important that we embrace new technology because that’s the way of the future. …we have to embrace it and fully embrace it. I want to get on with it.” (P5)*
Technology self-efficacy “I know how to use all this stuff.”	*“I have three computers, three laptops my phone, tablets…I know how to use all this stuff.” (P3)* *“…I use an insulin pump…I’ve got laptop and desktop and that sort of stuff.” (P4)*
Performance Expectancy: Who is Responsible? Locus of responsibility.	*“…My only concern now is if there’s a real problem, my podiatry people turn around and say go to see the podiatrist at the ED.” (P1)* *“…before our appointment with the podiatrist they (should) get a summary of what’s happened since the last time they saw us.” (P5)* *“…have him or her (GP) say you need to see the podiatrist…No! You need to see the emergency people at the hospital.” (P1)* *“…there’s time constraints and things like that with GPs checking up, you might think ‘oh well, they’re seeing what I’m seeing on their… on my device,’ but they’re not looking at it until you raise it.” (P4)*
Effort expectancy “is it going to be constantly making noise?” Concerns about technology intrusiveness and burden.	*“…is it gonna be constantly making noise, like to your phone…it could get annoying after a while, and …human nature…if they start getting annoyed by something, you stop liking it or caring about it.” (P3)* “*..I sort of see it more as a tool to use, not as something that I would possibly use every day, or wonna use every day. …because some things can become a bit of a grind. You know, like you don’t do your blood sugars because it is just a pain in the arse.” (P4)*
Additional issues of durability, cost and accessibility.	*“My main concern … how long does this (insole) last? How long do you have to replace it? On the cost side of things, that would be a big factor for me ‘cause one, I’m broke and two I can’t justify spending money on stuff that I feel I wouldn’t need personally.”* (P3) *“..I’ve got innersoles in my shoes and they’re quite expensive to get made…but they will last to the end of time.” (P4)* *“Because I know it puts a lot of people off, the cost of insoles….we pay a fair wack for orthotics as it is?” (P5)* *“…maybe it needs to be looked into being subsidised by the government simply because we keep hearing amputations are on the rise…so what is it costing them…as against a subsidy for something like this…for people like us?” P2* *“..having these (insoles) available for specific clients …for 6 months, 3 months…so you’re not wearing it as an ongoing thing…(the insole is) paid for and is owned by the hospital and at the end of a certain period you just give it back.” (P2)*

#### *Attitude towards technology* “Bring it on!”

Most participants expressed positive attitudes towards trying a smart insole, which was motivated by expectations that it would assist them to monitor their feet, and prevent foot ulcerations and amputation (performance expectancy) (Table 4). The majority of participants believed that a smart insole would be valuable for prevention of foot complications, such as ulceration (Table 4).

#### “I know how to use all this stuff” technology self-efficacy

Most participants reported high levels of technological self-efficacy. They identified that technology was easily accessible to them, and they were confident in their ability to use a smart insole (Table 4). While participants reported high self-efficacy with technology and with the idea of a smart insole, they queried how they would interact with the device and how the device would function (performance expectancy and effort expectancy) (Table 4).

#### Performance expectancy: who is responsible? Locus of responsibility

Participants expressed concern about who should receive alerts from a smart insole, and in what circumstances (performance expectancy and effort expectancy). Some participants felt that their general practitioners should receive the alerts and act as ‘gatekeepers’, providing direction to the patient about how to respond and where to go for help (Table 4).

Participants also identified that smart insole summary data should be provided to their podiatrist prior to appointments, particularly if the process was automated (Table 4). They did, however, acknowledge that health and medical professionals were busy and may not have time to review large quantities of data from individual patients and provide timely advice (Table 4).

#### Effort expectancy “Is it going to be constantly making noise?” concerns about technology intrusiveness and burden

Participants were concerned that the device may become intrusive if alerts were provided too frequently (effort expectancy). One participant was concerned about notifications and noise (Table 4). Participants were concerned that a smart insole might add to their daily self-management burden (effort expectancy) (Table 4).

#### “How long will it last?” additional issues of durability, cost and accessibility

Additional issues that were raised by participants were durability and cost. Participants were concerned about the longevity of the smart insoles, and whether this would add to or replace existing footwear and orthotics (Table 4).

Participants suggested financial issues should be addressed through government-subsidies, or possibly some kind of technology loan scheme, believing the device would prevent foot ulceration and hospitalisation (Table 4). Another option floated was to have health care organisations own the device, and issue them to high-risk patients for temporary use for added monitoring (Table 4).

## Discussion

For this regional Australian population with diabetes, the combined interaction between the psychosocial domains of attitude, self-efficacy, performance expectancy and effort expectancy produced a model that significantly explained behavioural intention to adopt a smart insole. It was unsurprising that self-efficacy and attitude would act as predictors of behavioural intention and both domains demonstrated significant individual and multivariate associations with behavioural intention. Interestingly, while effort expectancy and performance expectancy were important domains in the multivariate analysis they were not individually associated with behavioural intention. Instead they acted as influential moderators of the model due to their individual correlation with attitude and self-efficacy. These quantitative findings were further validated by the qualitative findings, which demonstrated the significance of attitude and self-efficacy on participants’ adoption intentions, predicated on their belief in the insole’s clinical efficacy (performance expectancy) and anticipated ease of use (effort expectancy).

Previous applications of the UTAUT in health settings found a differing interaction between these four domains, finding that effort expectancy and performance expectancy were predictors of behavioural intention, while attitude and self-efficacy were the moderators [16, 17, 19]. Our model’s divergence might be explained by the fact that participants were provided with a description of the smart insole concept but did not trial the technology. As such, they reported uncertainty regarding what to expect from such a device with respect to performance and effort, especially when these issues were explored during the qualitative phase. Attitude and self-efficacy beliefs are likely to play a significant role when a new technology is first presented to a person, but considerations of performance and effort are key considerations for successful device utilisation. This was borne out during a three-month trial of adherence to alert-based cues from a smart insole to encourage preventative foot-care behaviours reported by Najafi et al. [13]. Using the Technology Acceptance Model to understand user acceptance, the authors found that the participants tended to agree that the device was useful, the expected performance of the device was high and that it was a pleasant experience to use. Interestingly, this belief was stronger for those who interacted more with the device through receiving more alerts [13]. This contrasts with the concern by focus group participants in our study, who were concerned about frequent alerts becoming intrusive. The role of alerts or reminder features in improving adherence to diabetes self-care has been demonstrated by a number of other studies [8] and is a feature in biofeedback and monitoring technologies. This could be especially important for people with DFD, as evidence suggests that basic education has little impact on preventative foot-care behaviour without frequent reinforcement [7, 27]. For optimal use in the longer term, a balance will need to be struck between receiving frequent enough alerts to encourage use of a device and provide feedback and reinforcement, but not so frequent as to become intrusive to users.

Focus group participants expected their treating clinicians to engage with smart insole feedback to direct their decision-making. The engagement of a patient’s clinical team with health monitoring devices is an important factor in supporting or discouraging adoption [11]. While clinicians’ engagement can influence technology adoption and utilisation, this engagement cannot practically extend to daily directions. Our findings indicate that the participants may have an external locus of control, as opposed to viewing a smart insole as a tool to increase their own autonomy and decision-making capacity in foot monitoring. Previous research in a similar population has shown that participant misperceptions about foot health contributes to poorer self-care [28, 29]. Therefore, educating and empowering patients to make autonomous decisions regarding technological feedback, thereby preventing dependence on clinicians, is essential.

Focus group participants identified device cost and durability as key concerns. This concern reflects the nature of public healthcare within Australia, where there is variable access to public funding for orthopaedic footwear and orthotics, which appears to be a major barrier to preventative foot-care services [30, 31]. Additionally, wearable foot-health biofeedback monitors will need to be compatible with custom-made orthotics and orthopaedic footwear to be a viable option for this population [30, 31].

### Limitations

Limitations of this study include that it drew from a small target population that might not represent the broader Australian rural population, limiting the generalizability of these findings, and the power of the regression analysis. Additionally, because this study aimed to explain behavioural intention rather than behaviour, the technology was not actually trialled by participants; consequently, the participants were not able to determine the usefulness and usability of a smart insole. Purposeful sampling was used to recruit a focus group, however the final sample was predominantly male.

## Conclusions

Using an established theoretical framework, the findings of this mixed-methods study demonstrate that attitude, self-efficacy, performance expectancy and effort expectancy combine to predict the intention of adults with diabetes from regional Australia to adopt smart insole technology. Clinicians should consider these key psychosocial factors when prescribing and implementing technologies with patients at high risk of foot ulceration.

## Additional file

Additional file 1: Modified Unified Theory of Acceptance and Use of Technology Questionnaire. (ZIP 45 kb)

## Abbreviations

UTAUT: Unified theory of acceptance and use of technology
