# Supplementary material for: Factors influencing behavioural intention to use a smart shoe insole in regionally based adults with diabetes: a mixed methods study
Source: J Foot Ankle Res. 2019 May 20;12:29. doi: 10.1186/s13047-019-0340-3 (PMC6528213; doi:10.1186/s13047-019-0340-3)
Supplement: Supplementary file 1 — Modified Unified Theory of Acceptance and Use of Technology Questionnaire. (ZIP 45 kb) [file 13047_2019_340_MOESM1_ESM.zip › Additional File 1_JFAR-D-19-00016_R1R2.docx]

| **Smart-Insole Telehealth Questionnaire** |
| --- |
| Dear participant,  La Trobe University is conducting research on the use of a smart-insole that is currently being developed. A smart-insole would be put into your shoe, and would contain sensors that measure a range of variables such as steps taken and pressure on different parts of your foot. The insole would communicate with your mobile phone wirelessly to provide you with feedback about your feet, and possibly warn you if there is a problem with your foot. It is hoped that this type of technology, when fully developed, could help to patients prevent foot ulcers.  This questionnaire aims to analyse your perceptions about using this type of technology to help you manage your foot health. Your responses will help craft recommendations regarding the design and development of this type of technology and how it is provided to patients in the future.  Participation in the questionnaire is totally voluntary and it is estimated to take between 15 – 20 minutes to complete. |
| **1. Demographics** |
| **1. Gender**   - **Male** - **Female** |
| **2. Age**  ­­­­­­­__________ Years |
| **3. Number of years diagnosed with diabetes**  ­­­­­­­­­__________ Years |
| **4. Type of Diabetes**   - Type 1 Diabetes - Type 2 Diabetes - Unsure |
| **5. Country of birth** |

| **6. Highest level of education**   - **a.** Primary School - **b.** Some High School - **c.** Completed High School - **d.** Trade Qualification - **e.** TAFE Certificate or Diploma - **f.** Bachelors Degree - **g.** Post-Graduate Degree. | |
| --- | --- |
| **7. Technology you currently use at home**   - **a.** Touch-screen mobile phone - **b.** Internet from a home computer, laptop - **c.** Internet via your mobile phone - **e.** Tablet computer - **f.** Landline telephone only | |
| **8. Have you had previous foot ulcers?**   - Yes - No | |
| **2. UTAUT Questions** | |
| 1. **Performance Expectancy**   Please rate your answers to indicate your level of agreement with the statements in the right hand column. Mark your response anywhere on the scale from 0 to 4, with **0** being Strongly Disagree and **4** being Strongly Agree. | |
| **a.** I would find smart-insole equipment useful in managing my health. | \|_____\|_____\|_____\|_____\|  0 1 2 3 4  Strongly Disagree Strongly Agree |
| **b.** Using smart insole equipment would enable me to accomplish health monitoring tasks more quickly. | \|_____\|_____\|_____\|_____\|  0 1 2 3 4  Strongly Disagree Strongly Agree |
| **c.** Using smart insole equipment would allow me to be more involved and productive in my health care. | \|_____\|_____\|_____\|_____\|  0 1 2 3 4  Strongly Disagree Strongly Agree |
| **d.** If I used the smart insole equipment, I would increase my chance of improving my health. | \|_____\|_____\|_____\|_____\|  0 1 2 3 4  Strongly Disagree Strongly Agree |
| 1. **Please explain why you feel a smart-insole would be either useful or not useful in managing your health status:** | |
| **3. Effort Expectancy** | |
| **a.** I expect my interaction with a smart insole will be clear and understandable. | \|_____\|_____\|_____\|_____\|  0 1 2 3 4  Strongly Disagree Strongly Agree |
| **b.** I expect to find the smart insole equipment easy to use. | \|_____\|_____\|_____\|_____\|  0 1 2 3 4  Strongly Disagree Strongly Agree |
| **c.** I expect learning to operate the smart insole equipment will be easy for me. | \|_____\|_____\|_____\|_____\|  0 1 2 3 4  Strongly Disagree Strongly Agree |
| **d.** I expect to become skilled at using, or it will be easy for me to use the smart insole. | \|_____\|_____\|_____\|_____\|  0 1 2 3 4  Strongly Disagree Strongly Agree |
| **4. Attitude** | |
| **a.** Using a smart insole is a good idea. | \|_____\|_____\|_____\|_____\|  0 1 2 3 4  Strongly Disagree Strongly Agree |
| **b.** Using smart insole equipment would make managing my health care more interesting. | \|_____\|_____\|_____\|_____\|  0 1 2 3 4  Strongly Disagree Strongly Agree |
| **c.** I would like working with smart insole equipment. | \|_____\|_____\|_____\|_____\|  0 1 2 3 4  Strongly Disagree Strongly Agree |
| **5. Social Influence** | |
| **a.** People who influence my behaviour think that I should use smart insole equipment. | \|_____\|_____\|_____\|_____\|  0 1 2 3 4  Strongly Disagree Strongly Agree |
| **b.** People who are important to me think that I should use smart insole equipment. | \|_____\|_____\|_____\|_____\|  0 1 2 3 4  Strongly Disagree Strongly Agree |
| **c.** The doctors and/or caregivers have been supportive in the use of smart insole equipment. | \|_____\|_____\|_____\|_____\|  0 1 2 3 4  Strongly Disagree Strongly Agree |
| **d.** In general, the doctor and/or caregiver have supported the use of smart insole equipment. | \|_____\|_____\|_____\|_____\|  0 1 2 3 4  Strongly Disagree Strongly Agree |
| **6. Facilitating Conditions** | |
| **a.** I have the physical and mental ability necessary to use smart insole equipment. | \|_____\|_____\|_____\|_____\|  0 1 2 3 4  Strongly Disagree Strongly Agree |
| **b.** I have the knowledge necessary to use smart insole equipment. | \|_____\|_____\|_____\|_____\|  0 1 2 3 4  Strongly Disagree Strongly Agree |
| **c.** Smart insole equipment is not better than other methods I use to manage my health care. | \|_____\|_____\|_____\|_____\|  0 1 2 3 4  Strongly Disagree Strongly Agree |
| **d.** Support is available for assistance with difficulties I have or may experience with smart insole equipment. | \|_____\|_____\|_____\|_____\|  0 1 2 3 4  Strongly Disagree Strongly Agree |
| **7. Self-efficacy** | |
| **a.** I could complete most tasks using smart insole equipment if there was no one around to assist me. | \|_____\|_____\|_____\|_____\|  0 1 2 3 4  Strongly Disagree Strongly Agree |
| **b.** I could complete most tasks using smart insole equipment if I could call someone for help if I got stuck. | \|_____\|_____\|_____\|_____\|  0 1 2 3 4  Strongly Disagree Strongly Agree |
| **c.** I could complete most tasks using smart insole equipment with just the instructions provided me. | \|_____\|_____\|_____\|_____\|  0 1 2 3 4  Strongly Disagree Strongly Agree |
| **8. Anxiety** | |
| **a.** I feel nervous about using smart insole equipment. | \|_____\|_____\|_____\|_____\|  0 1 2 3 4  Strongly Disagree Strongly Agree |
| **b.** I worry that if I hit the wrong button my information may not be collected. | \|_____\|_____\|_____\|_____\|  0 1 2 3 4  Strongly Disagree Strongly Agree |
| **c.** I would hesitate to use smart insole equipment for fear of making mistakes that I cannot correct. | \|_____\|_____\|_____\|_____\|  0 1 2 3 4  Strongly Disagree Strongly Agree |
| **d.** Smart insole equipment would be somewhat intimidating to me. | \|_____\|_____\|_____\|_____\|  0 1 2 3 4  Strongly Disagree Strongly Agree |
| If you feel nervous or anxious about smart insole equipment, please explain or provide comment. | |
| **9. Behavioural Influences** | |
| **a.** I intend to use smart insole equipment in the next 365 days. | \|_____\|_____\|_____\|_____\|  0 1 2 3 4  Strongly Disagree Strongly Agree |
| **b.** I predict I will use smart insole equipment for the next 365 days. | \|_____\|_____\|_____\|_____\|  0 1 2 3 4  Strongly Disagree Strongly Agree |
| **c.** I plan to use smart insole equipment in the next 365 days. | \|_____\|_____\|_____\|_____\|  0 1 2 3 4  Strongly Disagree Strongly Agree |
| **10. What is the reason you don’t want to use smart insole equipment?** | |
| - **a.** Not interested - **b.** My family member/caregiver already does this for me - **c.** Will not help my current situation - **d**. I don’t want the responsibility for the equipment - **e.** Too much work - **f.** The equipment adds clutter to my house - **g.** Other, please specify - **f.** Not applicable | |
| **What might change your mind in accepting smart insole equipment?** | |
| - **a.** Nothing - **b.** Doctor/Nurse insists or encourages me to - **c.** Family Member wants me to - **d.** Home Health Care Nurse encourages me to due to another admission   to the hospital.   - **e.** Other (please specify) - **f.** Not applicable | |
