# Supplementary material for: Factors influencing behavioural intention to use a smart shoe insole in regionally based adults with diabetes: a mixed methods study
Source: J Foot Ankle Res. 2019 May 20;12:29. doi: 10.1186/s13047-019-0340-3 (PMC6528213; doi:10.1186/s13047-019-0340-3)
Supplement: Supplementary file 1 — Modified Unified Theory of Acceptance and Use of Technology Questionnaire. (ZIP 45 kb) [file 13047_2019_340_MOESM1_ESM.zip › Macdonald et al Additional File 1R2.docx]

**Additional File 1:** STROBE Statement.

|  | Item No | Recommendation |
| --- | --- | --- |
| **Title and abstract** | 1 | (*a*) Indicate the study’s design with a commonly used term in the title or the abstract  **Factors influencing behavioural intention to use a smart shoe insole in regionally based adults with diabetes: a mixed methods study** |
|  |  | (*b*) Provide in the abstract an informative and balanced summary of what was done and what was found. **Page 1-3** |
| Introduction | | |
| Background/rationale | 2 | Explain the scientific background and rationale for the investigation being reported **Page 4-5** |
| Objectives | 3 | State specific objectives, including any prespecified hypotheses **Page 6** |
| Methods | | |
| Study design | 4 | Present key elements of study design early in the paper **Page 6-9** |
| Setting | 5 | Describe the setting, locations, and relevant dates, including periods of recruitment, exposure, follow-up, and data collection **Page 6** |
| Participants | 6 | (*a*) *Cohort study*—Give the eligibility criteria, and the sources and methods of selection of participants. Describe methods of follow-up  **Page 6,8 Figure 1** |
|  |  | (*b*) *Cohort study*—For matched studies, give matching criteria and number of exposed and unexposed **NA** |
| Variables | 7 | Clearly define all outcomes, exposures, predictors, potential confounders, and effect modifiers. Give diagnostic criteria, if applicable **Page 6-9.** |
| Data sources/ measurement | 8* | For each variable of interest, give sources of data and details of methods of assessment (measurement). Describe comparability of assessment methods if there is more than one group. **Page 6-9** |
| Bias | 9 | Describe any efforts to address potential sources of bias **Page 6-8** |
| Study size | 10 | Explain how the study size was arrived at **Figure 1** |
| Quantitative variables | 11 | Explain how quantitative variables were handled in the analyses. If applicable, describe which groupings were chosen and why **Page 6-8** |
| Statistical methods | 12 | (*a*) Describe all statistical methods, including those used to control for confounding **Page 7,8** |
|  |  | (*b*) Describe any methods used to examine subgroups and interactions **Page 7,8** |
|  |  | (*c*) Explain how missing data were addressed **NA** |
|  |  | (*d*) *Cohort study*—If applicable, explain how loss to follow-up was addressed **NA**  *Case-control study*—If applicable, explain how matching of cases and controls was addressed **NA**  *Cross-sectional study*—If applicable, describe analytical methods taking account of sampling strategy **NA** |
|  |  | (*e*) Describe any sensitivity analyses **NA** |
| **Results** |  |  |
| Participants | 13* | (a) Report numbers of individuals at each stage of study—eg numbers potentially eligible, examined for eligibility, confirmed eligible, included in the study, completing follow-up, and analysed **Page 8-10 Figure 1** |
|  |  | (b) Give reasons for non-participation at each stage **Figure 1** |
|  |  | (c) Consider use of a flow diagram **Figure 1** |
| Descriptive data | 14* | (a) Give characteristics of study participants (eg demographic, clinical, social) and information on exposures and potential confounders **Table 2** |
|  |  | (b) Indicate number of participants with missing data for each variable of interest **NA** |
|  |  | (c) *Cohort study*—Summarise follow-up time (eg, average and total amount) **Page 6** |
| Outcome data | 15* | *Cohort study*—Report numbers of outcome events or summary measures over time |
|  |  | *Case-control study—*Report numbers in each exposure category, or summary measures of exposure **NA** |
|  |  | *Cross-sectional study—*Report numbers of outcome events or summary measures **NA** |
| Main results | 16 | (*a*) Give unadjusted estimates and, if applicable, confounder-adjusted estimates and their precision (eg, 95% confidence interval). Make clear which confounders were adjusted for and why they were included **Page 10-12, Tables 2,3,4** |
|  |  | (*b*) Report category boundaries when continuous variables were categorized **Page 8** |
|  |  | (*c*) If relevant, consider translating estimates of relative risk into absolute risk for a meaningful time period **NA** |
| Other analyses | 17 | Report other analyses done—eg analyses of subgroups and interactions, and sensitivity analyses **Page 12-14** |
| **Discussion** |  |  |
| Key results | 18 | Summarise key results with reference to study objectives **Page 15-17** |
| Limitations | 19 | Discuss limitations of the study, taking into account sources of potential bias or imprecision. Discuss both direction and magnitude of any potential bias **Page 17-18** |
| Interpretation | 20 | Give a cautious overall interpretation of results considering objectives, limitations, multiplicity of analyses, results from similar studies, and other relevant evidence **Page 15-17** |
| Generalisability | 21 | Discuss the generalisability (external validity) of the study results **Page 17-18** |
| **Other information** |  |  |
| Funding | 22 | Give the source of funding and the role of the funders for the present study and, if applicable, for the original study on which the present article is based **Page 19** |
